# Bogdanov Map for Modelling a Phase-Conjugated Ring Resonator

**DOI:** 10.3390/e21040384

**Published:** 2019-04-10

**Authors:** Vicente Aboites, David Liceaga, Rider Jaimes-Reátegui, Juan Hugo García-López

**Affiliations:** 1 Centro de Investigaciones en Óptica, Loma del Bosque 115, 37150 León, Mexico; 2 División de Ciencias e Ingenierías, Universidad de Guanajuato, Loma del Bosque 107, 37150 León, Mexico; 3 Centro Universitario de los Lagos, Universidad de Guadalajara, Enrique Diaz de León 1144, Paseos de la Montaña, Lagos de Moreno, 47460 Jalisco, Mexico

**Keywords:** spatial dynamics, Bogdanov Map, chaos, laser, resonator

## Abstract

In this paper, we propose using paraxial matrix optics to describe a ring-phase conjugated resonator that includes an intracavity chaos-generating element; this allows the system to behave in phase space as a Bogdanov Map. Explicit expressions for the intracavity chaos-generating matrix elements were obtained. Furthermore, computer calculations for several parameter configurations were made; rich dynamic behavior among periodic orbits high periodicity and chaos were observed through bifurcation diagrams. These results confirm the direct dependence between the parameters present in the intracavity chaos-generating element.

## 1. Introduction

Matrix description of optical systems through ABCD matrices (Equation (8)) naturally produces iterative maps with rather complex dynamics. Several publications have dealt with the ABCD law and the iterative maps it produces. Belanger [1] has generalized the ABCD propagation law for optical systems Onciul [2], using the Kirchhoff integral, derives a generalized ABCD propagation law for general astigmatic Gaussian beams through misaligned optical systems, Bastiaans [3] shows under what condition the well-known ABCD law that can be applied to describe the propagation of one-dimensional Gaussian light through first-order optical systems (or ABCD systems) can be extended to more than one dimension; in the two-dimensional (or higher-dimensional) case, an ABCD law only holds for partially coherent Gaussian light for which the matrix of second-order moments of the Wigner distribution function is proportional to a symplectic matrix. Tian [4] presents an iterative method for simulating beam propagation in nonlinear media using Hamiltonian ray tracing in which the Wigner distribution function of the input beam is computed at the entrance plane, used as the initial condition for solving the Hamiltonian equation; he gives examples for the study of periodic self-focusing, spatial solitons and the Gaussian–Schell model in Kerr-effect media. Finally, Siegman [5] and Tarasov [6] shown how to describe a laser resonator with iterative matrix optics by ray propagation through cascaded optical elements. This kind of map has been successfully applied before to the description of laser beams within optical resonators. This treatment has been explored for several other maps, obtaining several chaos-generating intracavity elements that are based on the dynamical behavior from widely diverse maps, such as the Ikeda map [7], Standard map [8], Tinkerbell map [9,10,11], Duffing map [11,12], logistic map [13] and the Henón map [11,14]. Throughout this article the Bogdanov Map will be used to describe a ring-phase conjugated resonator, while the resultant iterative matrix system is analyzed. In the following Section 2, a quick derivation of the Bogdanov map is sketched following reference [15], then will convert our *two*-dimensional mapping into a theoretical optical element that will produce the same complex dynamical behavior as the Bogdanov map within a phase-conjugated ring resonator. To accomplish this, we introduce the ABCD matrix formalism that is commonly used in paraxial optics [16], allowing us to represent each optical component as a 2×2 matrix. Moving forward with the previously obtained results, finding what we call *Bogdanov beams*; these are beams that propagate within the resonator following dynamics of the Bogdanov map. In Section 3, we discuss the results obtained from numerical calculations displaying the rich dynamics of the system, as it is shown in the bifurcation diagrams as a function of the intracavity chaos-generating element parameters. Finally, Section 4 presents the conclusions.

## 2. Material and Methods

### 2.1. Bogdanov Map

This map was originally conceived by Bogdanov while studying the universal unfolding of the double-zero-eigenvalue singularity [17] (also called *Bogdanov–Takens* or *cusp*), which is the equivalent of a vector field invariant under a rotation of the plane by *2π*. The Bogdanov map can be obtained by means of discretization using the Euler method on the Bogdanov vector field. Next, to be thorough and closely follow reference [15], we proceed to sketch a quick derivation of the Bogdanov Map.

(1) $$\begin{array}{c} \dot{y}=\theta ,\\ \dot{\theta }=0 \end{array}$$

This vector field has a codimension-two fixed point at the origin, known as a double-zero-eigenvalue singularity; the normal form of this can be written as follows:

(2) $$\begin{array}{cc} \dot{y} & =\theta +\lambda y^{2},\\ \dot{\theta } & =\eta y^{2} \end{array}$$

where λ≠0, η≠0. A two-parameter *versal* unfolding for this normal form, which contains all possible qualitative dynamical behavior near Equation (2), can be given:

(3) $$\begin{array}{cc} \dot{y} & =\theta +v_{2}y+\lambda y^{2},\\ \dot{\theta } & =v_{1}+\eta y^{2} \end{array}$$

The unfolding given above is not unique and a versal unfolding or deformation such as Equation (3) contains all possible qualitative dynamical behavior that can occur near the singularity. By restricting our attention to the region away from the saddle-node bifurcations, the Hamiltonian system of ordinary equations first considered by Bogdanov can be obtained,

(4) $$\begin{array}{cc} \dot{y} & =\theta \\ \dot{\theta } & =y(y-1) \end{array}$$

once again, a two-parameter versal unfolding is obtained for Equation (4),

(5) $$\begin{array}{cc} \dot{y} & =\theta \\ \dot{\theta } & =u_{1}\theta +y\left(y-1\right)+u_{2}y\theta \Xi \left(y,u_{1},u_{2}\right)+u_{2}^{2}\theta^{2}\Phi \left(y,\theta ,u_{1},u_{2}\right) \end{array}$$

By taking the vector field from Equation (5), and applying the backward Euler discretization method to the first equation ($\dot{y}$) and the forward Euler method to the second equation ($\dot{\theta }$), both with step length *h*, we obtain

(6) $$\begin{array}{cc} y_{n+1}= & y_{n}+h\theta_{n+1}\\ \theta_{n+1}= & \theta_{n}+hu_{1}\theta_{n}+hy_{n}\left(y_{n}-1\right)+hu_{2}y_{n}\theta_{n}\Xi \left(y_{n},u_{1},u_{2}\right)+hu_{2}^{2}\theta_{n}^{2}\Phi \left(y_{n},\theta_{n},u_{1},u_{2}\right) \end{array}$$

now making $\Xi (y_{n},u_{1},u_{2})=1$, $\Phi (y_{n},\theta ,u_{1},u_{2})=0$ and multiplying the second equation by *h*. Finally, making the change of variables $u_{1}=\varepsilon /h$, $u_{2}=\mu /h$, $h\theta =\tilde{\theta }$, $h^{2}=k$ and dropping the tilde from θ, we get the Bogdanov Map.

(7) $$\begin{array}{cc} y_{n+1}= & y_{n}+\theta_{n+1}\\ \theta_{n+1}= & \theta_{n}+\varepsilon \theta_{n}+ky_{n}\left(y_{n}-1\right)+\mu y_{n}\theta_{n} \end{array}$$

The Bogdanov map is a planar quadratic map, conjugate to the Hénon-area-preserving map in its conservative limit (ε=μ=0). Here, ε and μ are related to the Bogdanov vector field, while *k* plays the role of step length in the discretization, such that for a small *k*, the map behavior will resemble the original vector field. The dissipative Hopf parameter ε determines the birth and growth from the origin for the primary Hopf invariant circle; the stability of this circle is determined by μ, while the Hamiltonian discretization parameter *k* determines the birth and growth of the island chains.

### 2.2. Paraxial Matrix Analysis

The description of ray or Gaussian optics with matrices turns both the analysis and composition of optical systems into a simple and straightforward task, since this technique allows us to represent the behavior of any optical element as a 2×2 matrix. Cylindrical symmetry is used around the optical axis, so that for any given position *z* both the perpendicular distance of any ray to the optical axis (*y*) and its angle with the same axis (θ) can be defined; thus, any optical system can be represented by an [ABCD] matrix,

(8) $$\left(\begin{array}{c} y_{n+1}\\ \theta_{n+1} \end{array}\right)=\left(\begin{array}{cc} A & B\\ C & D \end{array}\right)\left(\begin{array}{c} y_{n}\\ \theta_{n} \end{array}\right)$$

In passive optical elements (mirrors, lenses, interfaces between two media, etc.), elements A,B,C,D are constant; nevertheless, for nonlinear optical elements, they are not necessarily constant, but may be functions of different parameters; The description of an optical system described by a Bogdanov Map requires (from Equation (7)) that the coefficients A,B,C,D be:

(9) $$\left(\begin{array}{cc} A & B\\ C & D \end{array}\right)=\left(\begin{array}{cc} 1 & \frac{\theta_{n+1}}{\theta_{n}}\\ k(y_{n}-1) & 1+\varepsilon +\mu y_{n} \end{array}\right)$$

where the value $\frac{\theta_{n+1}}{\theta_{n}}$ can be written as

$$\frac{\theta_{n+1}}{\theta_{n}}\equiv 1+\varepsilon +y_{n}\left[\frac{k}{\theta_{n}}\left(y_{n}-1\right)+\mu \right]$$

**Figure 1:**
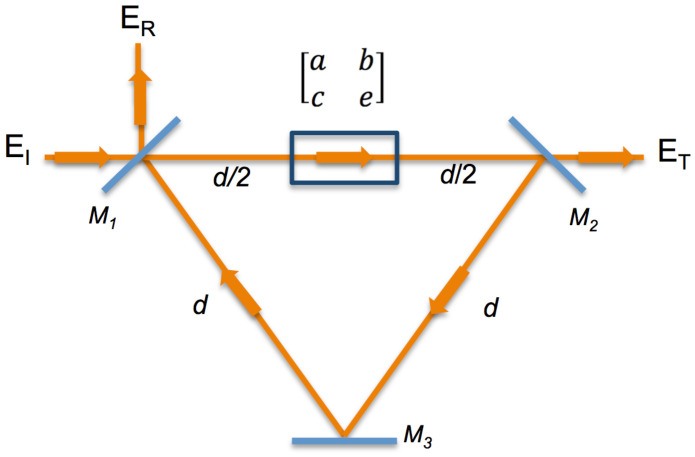
Phase-conjugated ring resonator with an intracavity chaos-generating element.

In Figure 1, we sketch the diagram of our optical system, where the [a,b,c,e] matrix is the unknown map generating device, located between the plain mirrors $M_{1}$ and $M_{2}$ at a distance d/2, while $M_{3}$ is a phase-conjugated mirror. For this system, the total transformation [ABCD] matrix for a complete round trip is written as follows:

(10) $$\begin{array}{cc} \left(\begin{array}{cc} A & B\\ C & D \end{array}\right) & =\left(\begin{array}{cc} 1 & 0\\ 0 & -1 \end{array}\right)\left(\begin{array}{cc} 1 & d\\ 0 & 1 \end{array}\right)\left(\begin{array}{cc} 1 & 0\\ 0 & 1 \end{array}\right)\left(\begin{array}{cc} 1 & d/2\\ 0 & 1 \end{array}\right)\\ & \times \left(\begin{array}{cc} a & b\\ c & e \end{array}\right)\left(\begin{array}{cc} 1 & d/2\\ 0 & 1 \end{array}\right)\left(\begin{array}{cc} 1 & 0\\ 0 & 1 \end{array}\right)\left(\begin{array}{cc} 1 & d\\ 0 & 1 \end{array}\right) \end{array}$$

which gives

(11) $$\begin{array}{cc} & =\left(\begin{array}{cc} a+\frac{3cd}{2} & \;b+\frac{3d}{4}\left(2a+3cd+2e\right)\\ -c & -\frac{3cd}{2}-e \end{array}\right) \end{array}$$

$$\begin{array}{cc} A & =a+\frac{3cd}{2}\\ B & =\;b+\frac{3d}{4}\left(2a+3cd+2e\right)\\ C & =-c\\ D & =-\frac{3cd}{2}-e \end{array}$$

To reproduce the behavior of the Bogdanov map by means of a ray within the optical ring resonator, each round trip described by $\left(y_{n},\theta_{n}\right)$ must be considered as an iteration of the Bogdanov map. Next, we take the previously obtained [ABCD] matrix elements of the Bogdanov map, Equation (9), and equate them to the total [ABCD] matrix of the resonator, Equation (11); this in order to generate the round-trip map dynamics for $\left(y_{n+1},\theta_{n+1}\right)$. Note here that the results obtained are only valid for a small *b* value, (b≈0): this is because before and after the matrix element [a,b,c,e], there is a propagation of (d−b)/2. Meanwhile, for a general case, Equation (11) ought to be replaced by the following:

(12) $$\begin{array}{cc} \left(\begin{array}{cc} A & B\\ C & D \end{array}\right) & =\left(\begin{array}{cc} 1 & 0\\ 0 & -1 \end{array}\right)\left(\begin{array}{cc} 1 & d\\ 0 & 1 \end{array}\right)\left(\begin{array}{cc} 1 & 0\\ 0 & 1 \end{array}\right)\left(\begin{array}{cc} 1 & \frac{d-b}{2}\\ 0 & 1 \end{array}\right)\\ & \times \left(\begin{array}{cc} a & b\\ c & e \end{array}\right)\left(\begin{array}{cc} 1 & \frac{d-b}{2}\\ 0 & 1 \end{array}\right)\left(\begin{array}{cc} 1 & 0\\ 0 & 1 \end{array}\right)\left(\begin{array}{cc} 1 & d\\ 0 & 1 \end{array}\right) \end{array}$$

which gives

(13) $$\left(\begin{array}{cc} a-\frac{c}{2}\left(b-3d\right) & \;\frac{1}{4}\left[b^{2}c-2b\left(-2+a+3cd+e\right)+3d\left(2a+3cd+2e\right)\right]\\ -c & \frac{1}{2}\left(bc-3cd-2e\right) \end{array}\right)$$

$$\begin{array}{cc} A & =a-\frac{c}{2}\left(b-3d\right)\\ B & =\;\frac{1}{4}\left[b^{2}c-2b\left(-2+a+3cd+e\right)+3d\left(2a+3cd+2e\right)\right]\\ C & =-c\\ D & =\frac{1}{2}\left(bc-3cd-2e\right) \end{array}$$

This is the total round-trip transformation matrix for the general case.

### 2.3. Bogdanov Beams

We define ‘Bogdanov beams’ as beams that behave on the $y_{n}$ and $\theta_{n}$ optical ray parameters according to the Bogdanov Map given by Equation (7), i.e., Beams produced in the above optical resonator that undergo the Bogdanov map dynamics will be called ‘Bogdanov beams’. To obtain the Bogdanov beams, the matrix elements of Equation (9) must be equaled to the elements of Equation (11), thus giving the system.

(14) $$\begin{array}{cc} a+\frac{3cd}{2} & =1\\ b+\frac{3d}{4}\left(2a+3cd+2e\right) & =1+\varepsilon +y_{n}\left[\frac{k}{\theta_{n}}\left(y_{n}-1\right)+\mu \right]\\ -c & =k(y_{n}-1)\\ -\frac{3cd}{2}-e & =1+\varepsilon +\mu y_{n} \end{array}$$

This system is solved to obtain the [a,b,c,e] matrix elements. Therefore, the intracavity matrix that produces Bogdanov Beams is

(15) $$\left(\begin{array}{cc} a & b\\ c & e \end{array}\right)=\left(\begin{array}{cc} \left[1+\frac{3}{2}kd\left(y_{n}-1\right)\right] & \;\frac{\theta_{n+1}}{\theta_{n}}-\frac{3}{2}d\left\{\frac{3}{2}kd\left(y_{n}-1\right)-\varepsilon -\mu y_{n}\right\}\\ -k(y_{n}-1) & -[1+\varepsilon +\mu y_{n}+\frac{3}{2}kd\left(y_{n}-1\right)] \end{array}\right)$$

### 2.4. General Case for Bogdanov Beams

Taking the elements of matrix Equation (9) and equating them to the ones of matrix Equation (13), we get the following system, which is analogous to Equation (14):

(16) $$\begin{array}{cc} a-\frac{c}{2}\left(b-3d\right) & =1\\ \frac{1}{4}\left[b^{2}c-2b\alpha +3d\beta \right] & =1+\varepsilon +y_{n}\left[\frac{k}{\theta_{n}}\left(y_{n}-1\right)+\mu \right]\\ -c & =k(y_{n}-1)\\ \frac{1}{2}\left(bc-3cd-2e\right) & =1+\varepsilon +\mu y_{n} \end{array}$$

Here α=(−2+a+3cd+e) and β=(2a+3cd+2e).

Solving the system found in Equation (16), we find two new [a,b,c,e] matrices, Equations (17) and (18). These matrices contain all the dynamic information of the Bogdanov map taking into account the thickness *b* of the intracavity element,

(17) $$\left(\begin{array}{cc} a & b\\ c & e \end{array}\right)=\left(\begin{array}{cc} \frac{1}{6\theta_{n}}\left(\vartheta_{n}-\gamma_{n}\right) & \frac{1}{3k\theta_{n}\left(y_{n}-1\right)}\left(\varphi_{n}+\gamma_{n}\right)\\ k(1-y_{n}) & \frac{1}{6\theta_{n}}\left(\varrho_{n}-\gamma_{n}\right) \end{array}\right)$$

(18) $$\left(\begin{array}{cc} a & b\\ c & e \end{array}\right)=\left(\begin{array}{cc} \frac{1}{6\theta_{n}}\left(\vartheta_{n}+\gamma_{n}\right) & \frac{1}{3k\theta_{n}\left(y_{n}-1\right)}\left(\varphi_{n}-\gamma_{n}\right)\\ k(1-y_{n}) & \frac{1}{6\theta_{n}}\left(\varrho_{n}+\gamma_{n}\right) \end{array}\right)$$

were γ,ϑ,φ,ϱ are defined as:

$$\begin{array}{c} \gamma_{n}\equiv \{\theta_{n}[-12k^{2}{\left(y_{n}-1\right)}^{2}y_{n}+\theta_{n}[36k^{2}d^{2}{\left(y_{n}-1\right)}^{2}+{\left(2+\varepsilon +\mu y_{n}\right)}^{2}\\ -12k\left(y_{n}-1\right)\left(1+\varepsilon +\mu y_{n}+d\left(-1+\varepsilon +\mu y_{n}\right)\right){]]\}}^{1/2} \end{array}$$

$$\begin{array}{c} \vartheta_{n}\equiv \theta_{n}\left(8+\varepsilon +12kd\left(y_{n}-1\right)+\mu y_{n}\right) \end{array}$$

$$\begin{array}{c} \varphi_{n}\equiv -\theta_{n}\left(2+\varepsilon +3kd\left(y_{n}-1\right)+\mu y_{n}\right) \end{array}$$

$$\begin{array}{c} \varrho_{n}\equiv \theta_{n}\left(-4-5\varepsilon +12kd\left(y_{n}-1\right)-5\mu y_{n}\right) \end{array}$$

the intracavity chaos-generating matrix, whose $b_{n}$ element is given as follows;

(19) $$b_{n}\equiv \frac{1}{3k\theta_{n}\left(y_{n}-1\right)}\left(\varphi_{n}-\gamma_{n}\right)$$

## 3. Results

### Computer Calculations

The dynamic behavior of the phase-conjugated resonator in phase space was studied through numerical iteration of the obtained matrices, Equations (17) and (18). To find valid trajectories on the phase plane values for $y_{n},\theta_{n}$ must be real numbers at every iteration, diverging trajectories are only mathematical possibilities since they cannot be related to any physical reality given that they do not meet the stability requirements to stay within the resonator. Also, the $b_{n}$ intracavity element from the matrices must be greater than zero at every iteration, while being smaller than the mirror resonator separation distance. These conditions ensure that the trajectories are on the real phase plane and within a stable trajectory, greater than zero at every iteration, given that the $b_{n}$ element is related to the total round-trip distance traveled by the Bogdanov beam within the resonator. The last condition ensures that no ‘negative distances’ are traveled.

**Figure 2:**
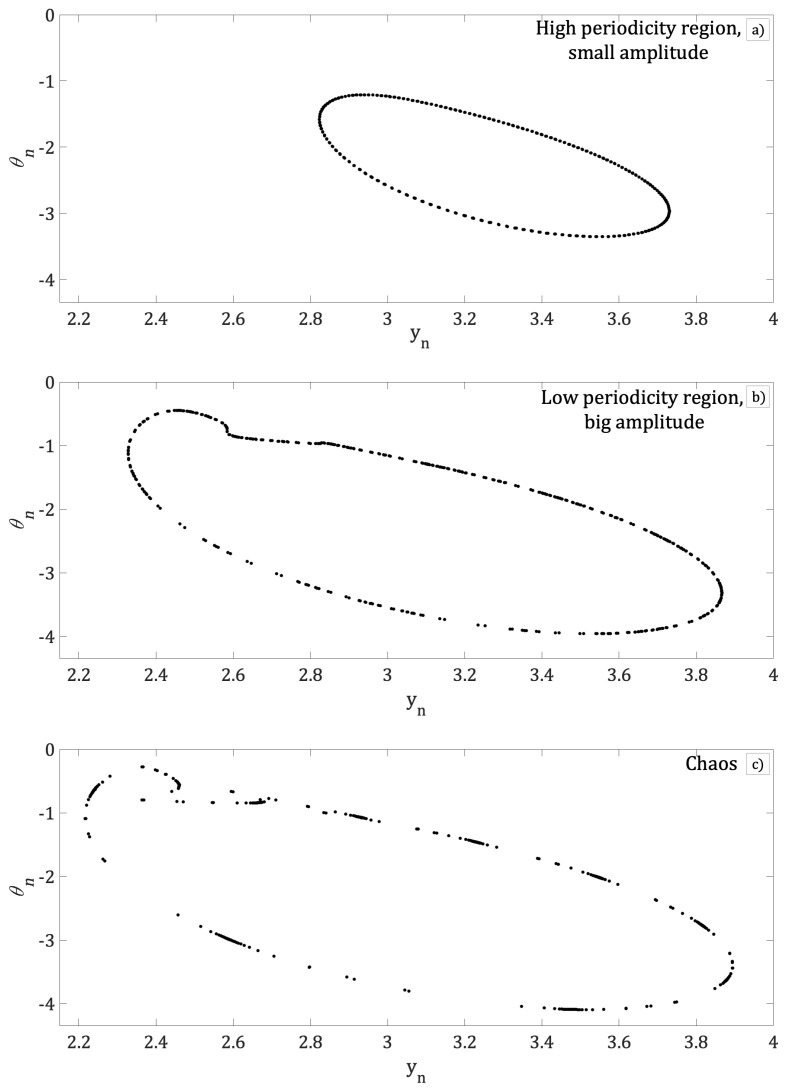
Phase space ($y_{n}$, $\theta_{n}$), equivalent to a round trip inside the resonator for (**a**) d=0.95, (**b**) d=0.99 and (**c**) d=0.998; in all cases k=0.295, ε=0.01 and μ=−0.1.

Iterations were carried out using Equation (18) for values of the control parameter *d*, where the iterations ($y_{n}$, $\theta_{n}$) have physical meaning. The system displays high periodicity for 0.91<d<1, Figure 2a. Also, a short region of low periodicity appears within a high periodicity range where d=0.99 Figure 2b. For d>0.9925, the Bogdanov beam resonator exhibits a period-doubling route to chaos, Figure 2c.

The bifurcation diagram of $b_{n}$ with was obtained to understand the dependence of the intra cavity nonlinear element $b_{n}$ with respect to parameters; *d*, *k* and ε of the Bogdanov map. Advantages of this bifurcation diagram is that it gives a global view of the dynamic element $b_{n}$ as one or several parameters are changed.

**Figure 3:**
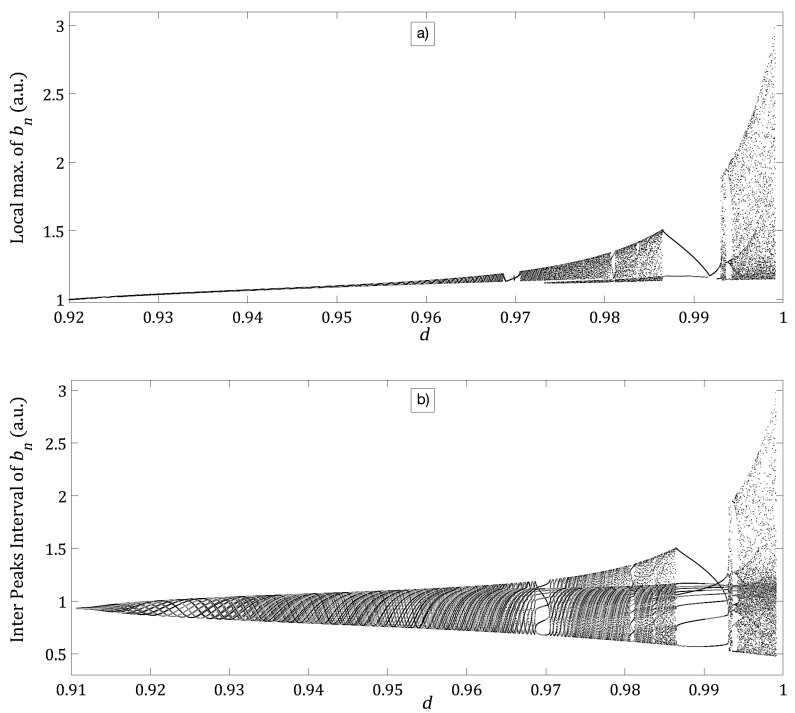
(**a**) Bifurcation diagram of local max of $b_{n}$ as a function of parameter *d*. (**b**) Temporal inter peak interval (IPI) of $b_{n}$ as a function of parameter *d*; in both plots, the following fixed values were used: k=0.295, ε=0.01 and μ=−0.1.

Figure 3a shows the bifurcation diagram of local max of $b_{n}$ as a function of parameter *d*. In this figure, high periodicity is interrupted by regions of low periodicity windows and a route to chaos by period-doubling is shown. The same result is also shown while plotting the temporal Inter Peak Intervals (IPI) of $b_{n}$ as the parameter *d* is varied, Figure 3b. Comparing Figure 3a and Figure 3b, it is shown that Figure 3b clearly illustrates a rich dynamics that shows high periodicity for 0.91<d<1, Figure 2a, interrupted by low periodicity windows of for d=0.99, Figure 2b. The bifurcation diagrams show a route to chaos due to period-doubling, Figure 2c.

**Figure 4:**
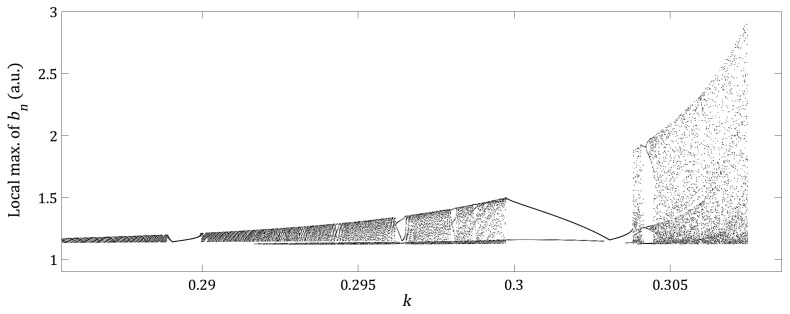
Bifurcation diagram of local max of $b_{n}$ as a function of parameter *k*, for d=0.9837, ε=0.01 and μ=−0.1.

**Figure 5:**
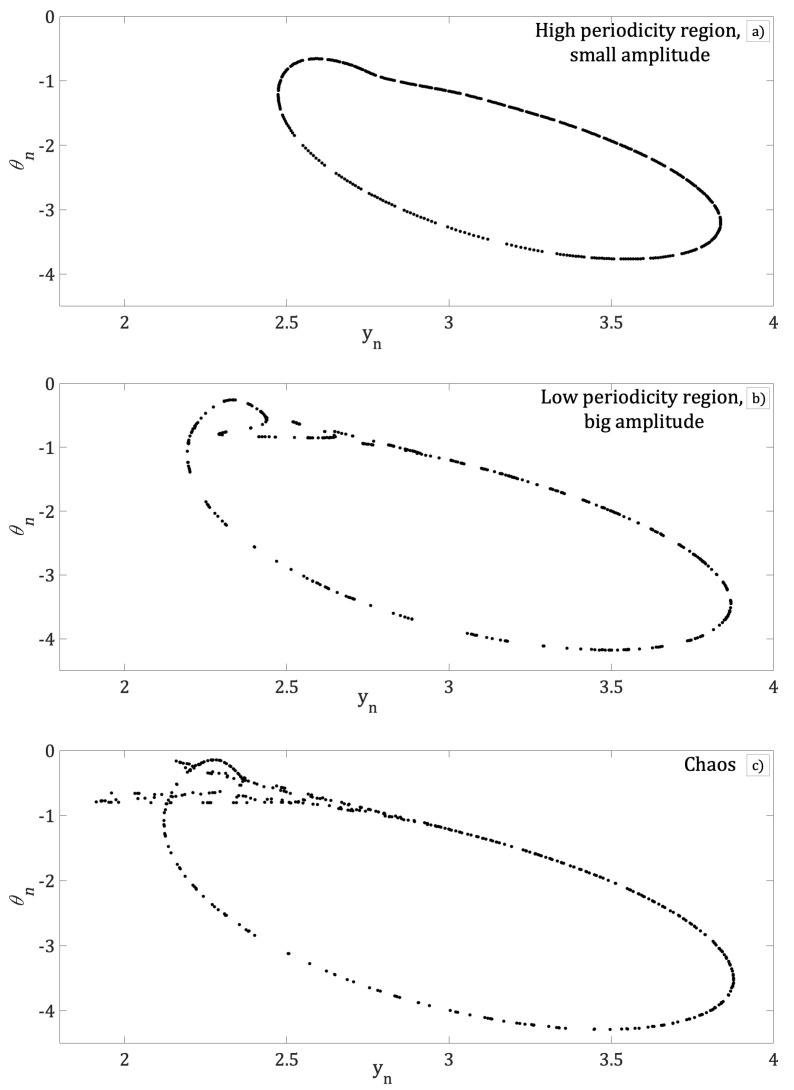
Phase space ($y_{n}$, $\theta_{n}$). High periodicity for (**a**) k=0.2925, low periodicity for (**b**) k=0.30434 and chaos for (**c**) k=30735, in all cases d=9837, ε=0.01 and μ=−0.1.

As can be seen, the dependence of the intra cavity nonlinear element $b_{n}$ to parameter *d* of the phase-conjugated ring resonator has been shown. In the following figures, dependence of $b_{n}$ on the *k* and ε parameters of the Bogdanov map will be displayed. Figure 4 shows the bifurcation diagram of the local max of $b_{n}$ as a function of parameter *k*. Although Figure 4 is qualitatively similar to Figure 3a clear difference is noted when the low periodicity windows are considered. It can be observed that when the parameter *k* is increased, the region of high periodicity is interrupted by windows with low values of periodicity, i.e., for k=0.2964, and for k=0.30735 exhibits a route to chaos by period-doubling. The phase space ($y_{n}$, $\theta_{n}$) for particularly cases of high and low periodicity and chaos is shown in Figure 5a–c respectively.

**Figure 6:**
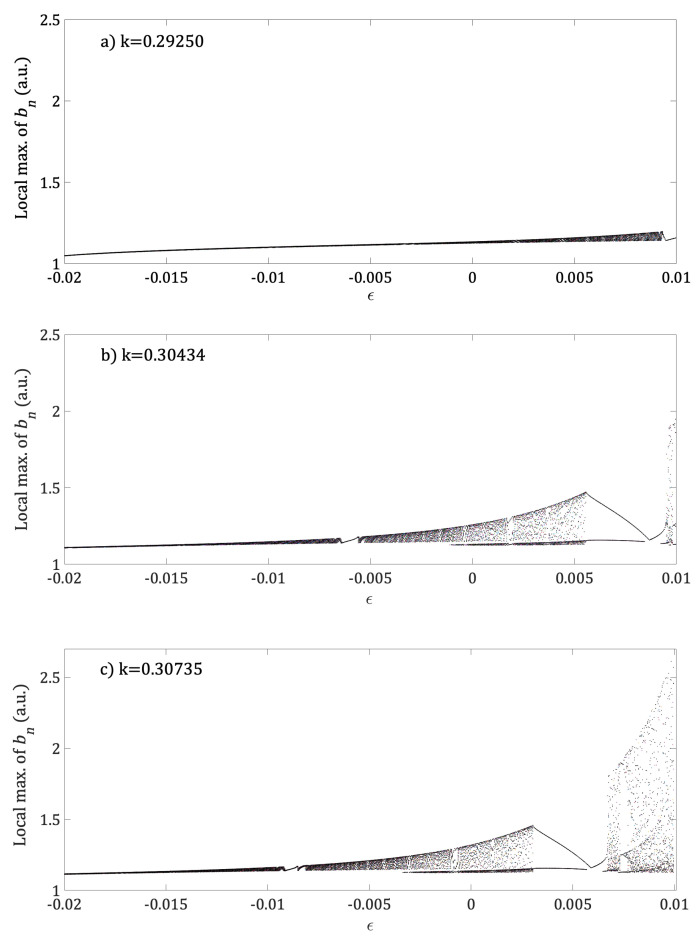
The bifurcation diagram of local max $b_{n}$ as a function of ε for three different values of *k* with *d* fixed in the chaotic region, and μ=−0.1. (**a**) k=0.2925, (**b**) k=0.30434 and (**c**) k=0.30735.

In addition, the phase space ($y_{n},\theta_{n}$) for different values of *k* with *d* fixed in chaotic region are plotted in Figure 5, while the bifurcation diagram of local max $b_{n}$ as a function of ε for same values of *k* and *d*, are plotted in Figure 6. In this figure, we can see that for k=0.2894, Figure 5a, the bifurcation diagram of local max $b_{n}$ presents a high periodicity for all the range of control parameter ε; see Figure 6a. With further increase of parameter *k* to values of k=0.30434, Figure 5b, the bifurcation diagram Figure 6b show a short interval of ε where the local max of $b_{n}$ exhibits low periodicity windows that interrupts a region of high periodicity. Finally, for k=0.30735 (chaotic region of Figure 5c), Figure 6c shows regions of high periodicity interrupted by low periodicity windows and a large region of route to chaos by period-doubling as control parameter ε is increased.

## 4. Conclusions

In this paper, a matrix transformation over the Bogdanov map is proposed to obtain an intra cavity element that can yield the same rich, dynamical behavior within a phase-conjugated ring resonator. We began our study by obtaining the Bogdanov Map through the use of Euler method for discretization over the Bogdanov Vector Field; then, we introduced the paraxial matrix analysis (or ABCD propagation law): this was done in order to simplify the analysis for the complete resonator system, enabling us to express this system as a simple dynamical matrix Equation (8). Once these central concepts had been introduced, we proceed to obtain what we call “Bogdanov Beams”, which are beams produced in an optical resonator undergoing the Bogdanov map dynamics. Then, we studied a simple case of ‘Bogdanov Beams’ where the thickness of the intra cavity element is considered to be negligible. Next, we moved on to the general case, where the thickness of the intracavity element is greater than zero. While it may seem a trivial difference, this general case introduces a new parameter *d* in our final matrix transformation, which adds up to the three initial parameters from the Bogdanov Map (k,ε,μ), therefore increasing the dimension of the problem and contributing to the non-linearity of the map. Once the explicit expressions for the general case were obtained, Equations (17) and (18), computer programs were made that allowed us to search the 4-dimensional parameter space for combinations that yield stable trajectories; this is no easy task, since the stability of the trajectories is also dependent on the initial values ($y_{0},\theta_{0}$), due to this, often the trajectories will not have physical meaning; it is important to remark that we analyzed valid intervals of the parameters (k,ε,μ and *d*). We have found that the intracavity element, $b_{n}$, Equation (19), is responsible for the different dynamic behavior of the optical resonator. The response of $b_{n}$ to the parameters (k,ε,μ and *d*) by bifurcation diagrams of local max and IPI of time series of $b_{n}$ has been accomplished.

The dependence of $b_{n}$ with respect to *d*, which is the distance between plain mirrors of the phase-conjugated ring resonator showed low, high periodicity and route to the chaos by period-doubling behavior, see Figure 3. Similar behavior was observed when the dependence of $b_{n}$ was analyzed with respect to the parameters k,ε while μ and *d* were fixed, see Figure 4. Interesting results were found for the dependence of $b_{n}$ on the parameter ε for different fixed values of *k*. For a small value of k=0.2925, the bifurcation diagram shows high periodicity of low amplitude, see Figure 6a. With an increment of k=0.30434, we have low periodicity windows within high periodicity regimens, see Figure 3b. Finally, at k=0.30735, the bifurcation diagram of local max of $b_{n}$, shows rich dynamics, with low and high periodicity regions and a route to chaos by period-doubling, see Figure 6c.

Based on the behavior observed, we conclude that the matrix transformation used was successful in generating a dynamical system that preserves the main structures found in the Bogdanov map. The practical implementation of an intracavity element is a complex technical challenge far beyond the aim of this work. Interested readers on this matter may consult reference [9].
